# Supplementary material for: Photochemical spin-state control of binding configuration for tailoring organic color center emission in carbon nanotubes
Source: Nat Commun. 2022 Aug 1;13:4439. doi: 10.1038/s41467-022-31921-0 (PMC9343348; doi:10.1038/s41467-022-31921-0)
Supplement: Supplementary file 1 — Supplementary Information [file 41467_2022_31921_MOESM1_ESM.pdf]

Supplementary Information for

Photochemical Spin-State Control of Binding Configuration for Tailoring Organic Color Center Emission in Carbon Nanotubes

Yu Zheng[1,*] Yulun Han[2], Braden M. Weight[1,2,3,4], Zhiwei Lin[5], Brendan J. Gifford[6], Ming Zheng[5], Dmitri Kilin[2], Svetlana Kilina[2], Stephen K. Doorn,[1] Han Htoon[1,§] and Sergei Tretiak[1,6,†]

[1]*Center for Integrated Nanotechnologies, Materials Physics and Applications Division, Los Alamos National Laboratory, NM 87545, United States*

[2]*Department of Chemistry and Biochemistry, North Dakota State University, Fargo, ND 58102, U.S.A.*

[3]*Department of Physics, North Dakota State University, Fargo, ND, 58102, U.S.A.*

[4]*Department of Physics and Astronomy, University of Rochester, Rochester, NY 14627, U.S.A*

[5]*Materials Science and Engineering Division, National Institute of Standards and Technology, Gaithersburg, MD 20899, U.S.A.*

[6]*Center for Nonlinear Studies, and Theoretical Division Los Alamos National Laboratory, Los Alamos, NM 87545, U.S.A.*

* email address: yz94rice@gmail.com

§ email address: htoon@lanl.gov

† email address: serg@lanl.gov

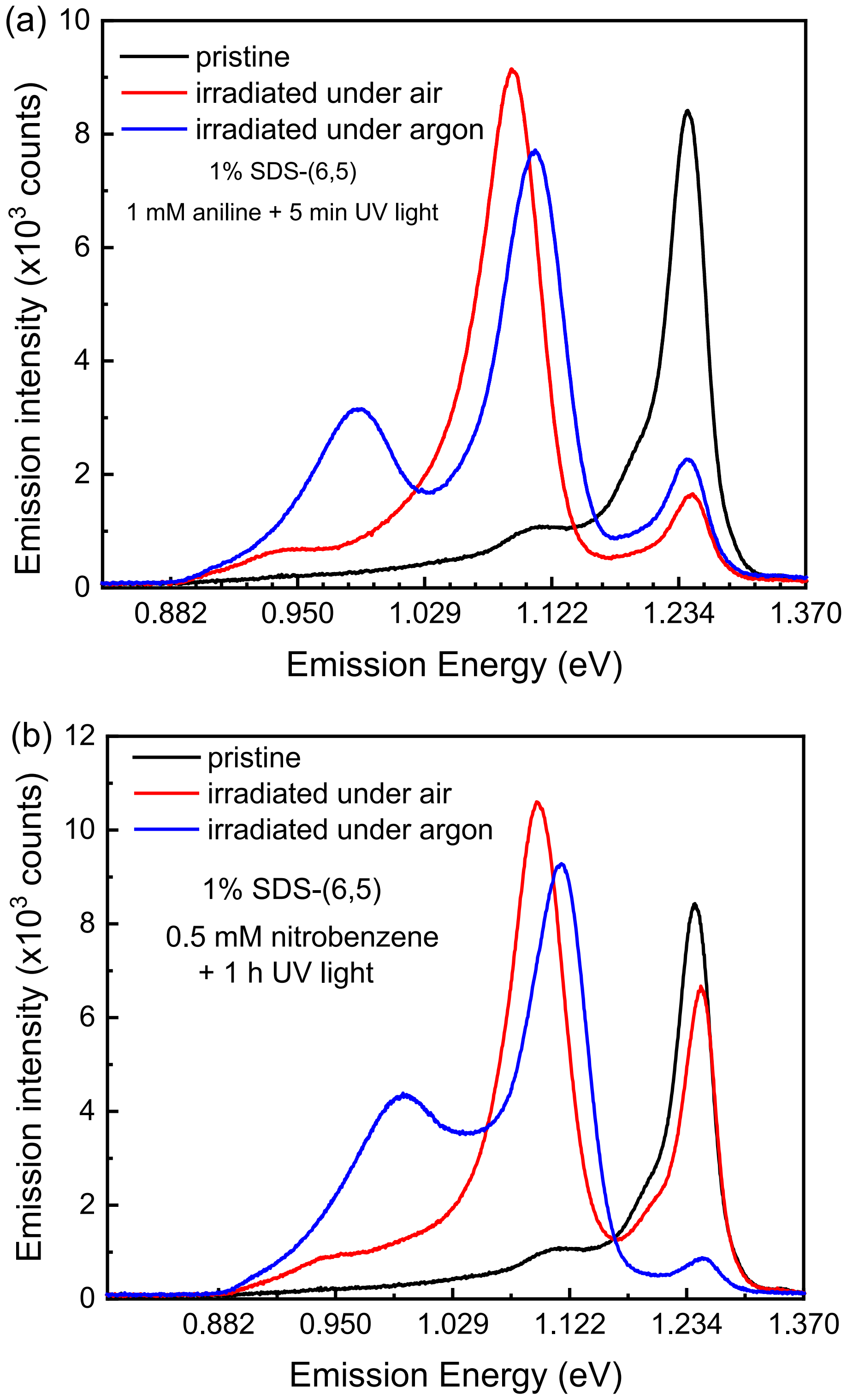

Supplementary Figure 1. (a) Photoluminescence spectra of pristine (6, 5) (black curve) and functionalized (6, 5) treated using 1 mM aniline under air (red curve) and argon (blue curve)

conditions. (b) Pristine (6, 5) and treated (6, 5) with 0.5 mM nitrobenzene under air and argon conditions.

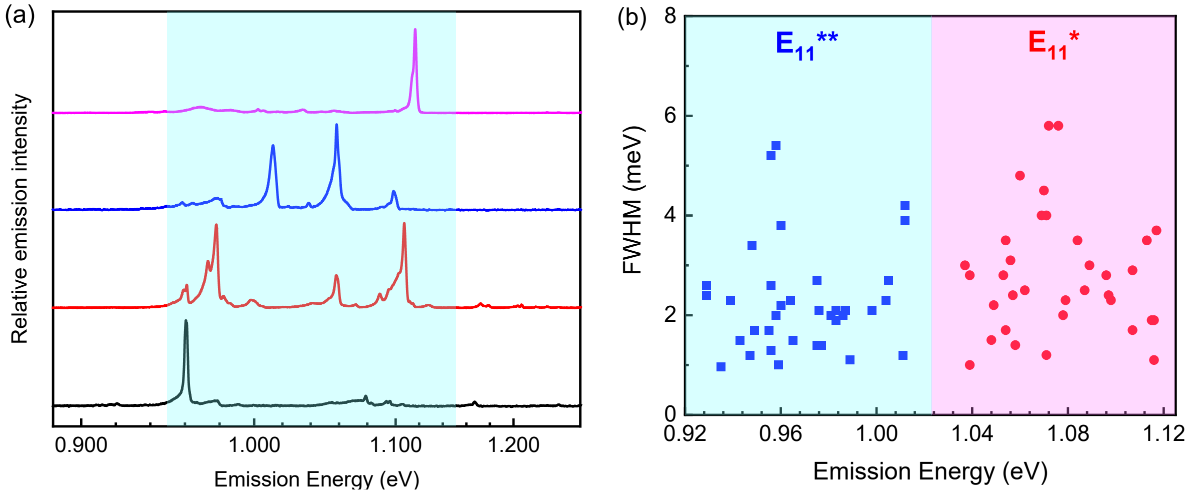

Supplementary Figure 2. (a) Low-temperature photoluminescence spectra (at ~ 4 K) for individual (6, 5) SWCNTs functionalized with the oxygen-free photoreaction. These spectra display three patterns including emission only in the 1100 – 1200 nm spectral range, emission only in the range of 1200 – 1300 nm, and multiple pronounced emission peaks in both ranges. (b) FWHM of E_{11}* and E_{11}** emission features for individual (6, 5) SWCNTs is plotted as a function of emission energy.

Supplementary Table 1. Peak position and full width at half maximum (FWHM) of emission for (6, 5) SWCNTs treated under air and argon conditions

<table>
<tr><th></th><th colspan="2">Peak position (eV)</th><th>FWHM (meV)</th></tr>
<tr><td>Treated in air</td><td colspan="2">1.062 ± 0.034</td><td>2.21 ± 1.19</td></tr>
<tr><td>Treated in Ar</td><td>0.969 ± 0.024</td><td>1.077 ± 0.024</td><td>2.61 ± 1.26</td></tr>
</table>

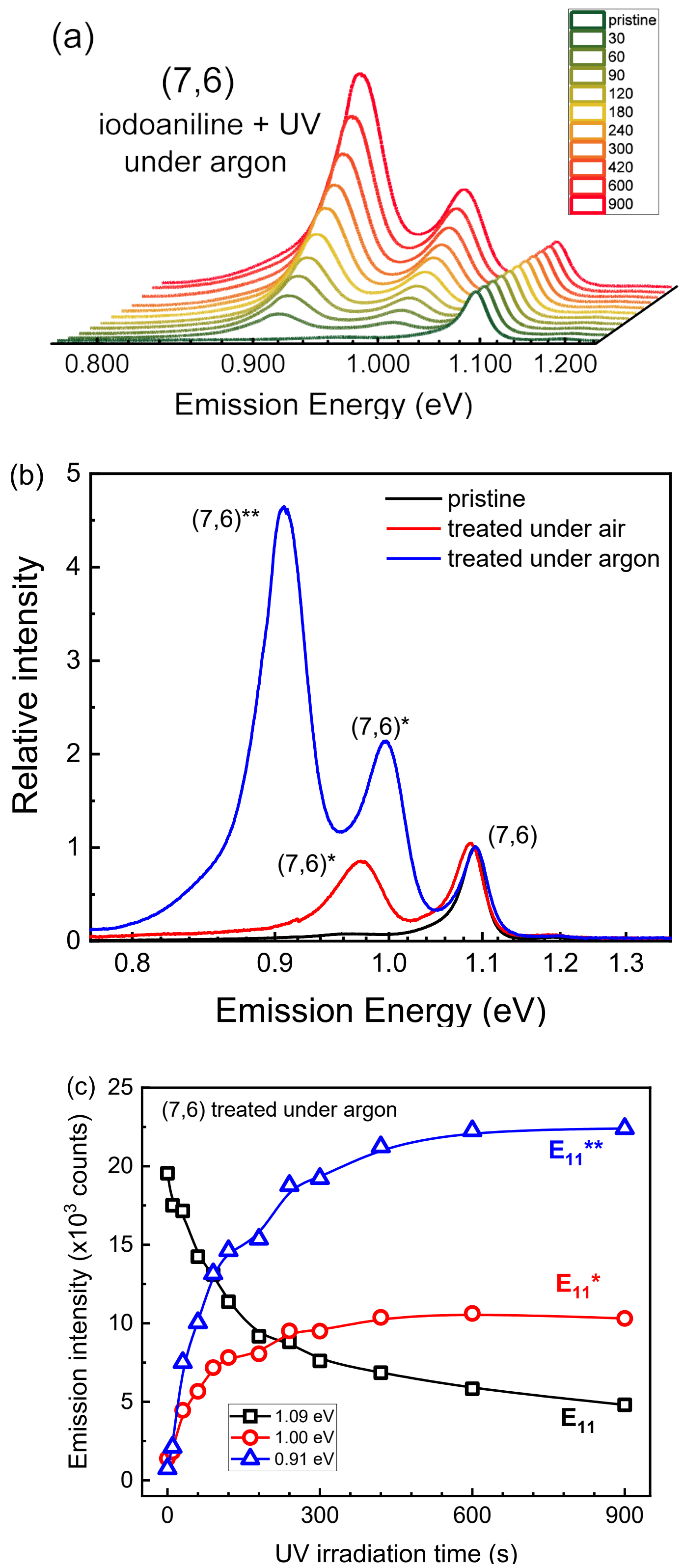

Supplementary Figure 3. (a) Photoluminescence spectra of (7, 6) SWCNTs treated under argon conditions with different UV irradiation times. (b) Emission spectra of pristine (7, 6) (black curve)

and functionalized (7, 6) treated using 0.63 mM iodoaniline under air (red curve) and argon (blue curve) conditions. (c) Intensities of E_{11}, E_{11}*, and E_{11}** emission are plotted as functions of UV irradiation time for (7, 6) SWCNTs treated in argon.

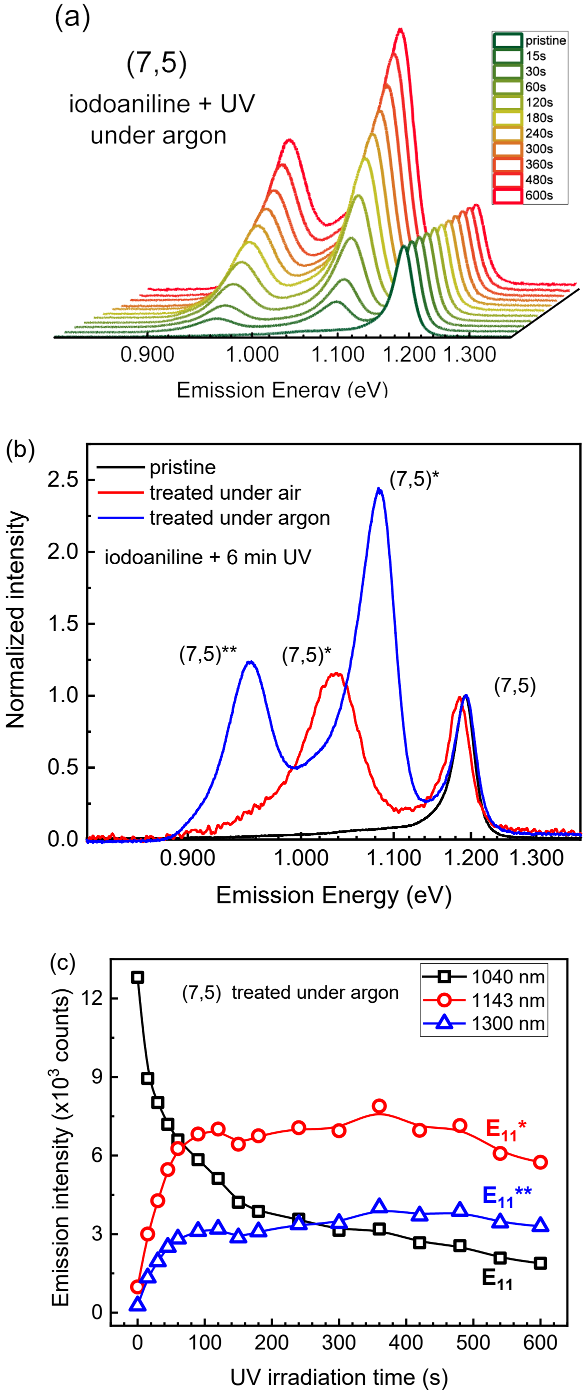

Supplementary Figure 4. (a) Photoluminescence spectra of (7, 5) SWCNTs treated under argon conditions with different UV irradiation times. (b) Emission spectra of pristine (7, 5) (black curve) and functionalized (7, 5) treated using 0.63 mM iodoaniline under air (red curve) and argon (blue curve) conditions. (c) Intensities of E_{11}, E_{11}*, and E_{11}** emission are plotted as functions of UV irradiation time for (7, 5) SWCNTs treated in argon.

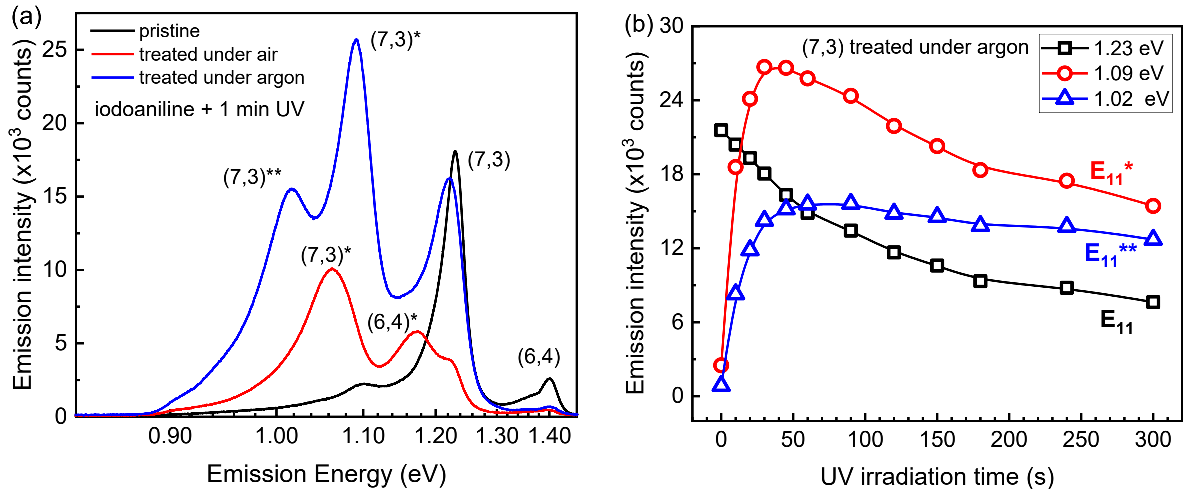

Supplementary Figure 5. (a) Emission spectra of pristine (7, 3) (black curve) and functionalized (7, 3) treated using 0.63 mM iodoaniline under air (red curve) and argon (blue curve) conditions. (b) Intensities of E_{11}, E_{11}*, and E_{11}** emission are plotted as functions of UV irradiation time for (7, 3) SWCNTs treated in argon.

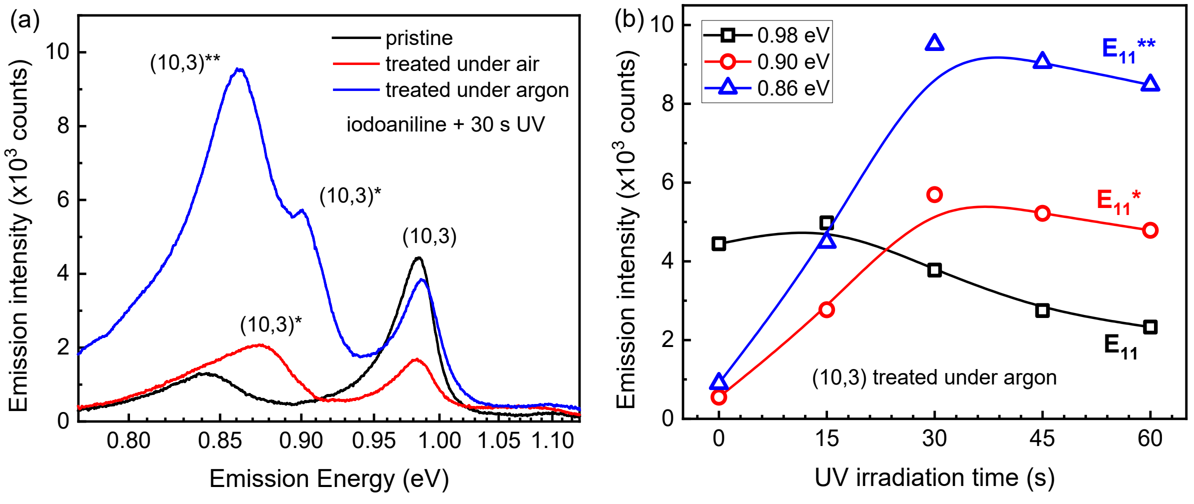

Supplementary Figure 6. (a) Emission spectra of pristine (10, 3) (black curve) and functionalized (10, 3) treated using 0.63 mM iodoaniline under air (red curve) and argon (blue curve) conditions. (b) Intensities of E_{11}, E_{11}*, and E_{11}** emission are plotted as functions of UV irradiation time for (10, 3) SWCNTs treated in argon.

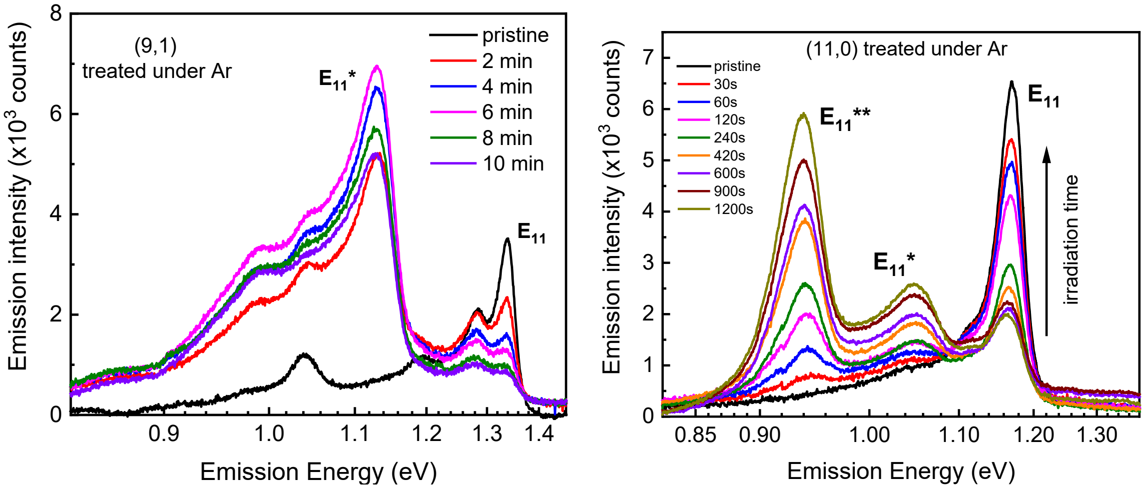

Supplementary Figure 7. Emission spectra of (9, 1) (left panel) and (11, 0) (right panel) SWCNTs treated using 0.63 mM iodoaniline under argon conditions with different UV irradiation times.

Supplementary Note 1

Analyses of spectroscopic features

The difference of ΔE_{11}* (air) and ΔE_{11}* (Ar) is between ca. 20 to 50 meV and shows some dependence on nanotube structure. We suspect that the small energy discrimination of E_{11}* emitting states obtained with / without oxygen arises from the different chemical identities of the second addend after the aryl group attachment. In the presence of oxygen, the –OH group could occupy the secondary reactive site, whereas the –H atom reacts with the secondary site in the absence of oxygen. This trend of the redshifted exciton of aryl-H compared to those of aryl-OH defect is consistent with the calculated HOMO-LUMO energy difference between the pristine and functionalized (6, 5) SWCNT by aryl-H and aryl-OH. Our theoretical findings show that the sp^3-defect created by aryl-H results in a smaller energy gap than those of aryl-OH by ca. 20 – 40 meV

depending on the methodology (see Supplementary Fig. 8). The similar linewidth of emission from E_{11}* and E_{11}** states whose energies are significantly different implies that the energy of exciton confinement at defect sites has negligible effect on the exciton dephasing, which contributes to the spectral linewidth (see Supplementary Fig. 7). The "mod 2" nanotube chiralities give slightly larger values of E_{11}* (Ar) – E_{11}* (air) as compared to the "mod 1" SWCNTs (see Supplementary Fig. 9b). The systematically slightly larger energy shifts (ca. 20 – 50 meV) of E_{11}* states for different nanotube chiralities obtained with air than that in argon indicates that the discrimination of E_{11}* obtained with / without oxygen likely arises from the different second addend species (–OH and –H, respectively), rather than from the different binding configurations of functional groups, since the latter has been shown to result in distinct defect-states with significantly different energies

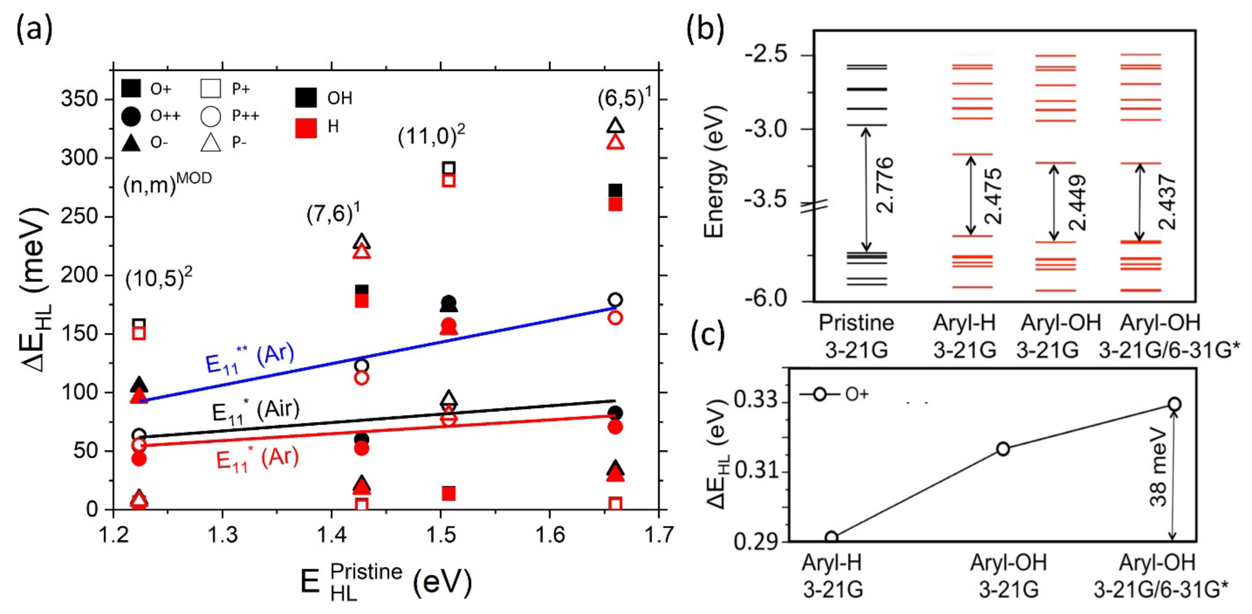

Supplementary Figure 8. (a) Similar to Fig. 3c, now including all defect configurations (symbol shape) and H/OH pairs (red/black). Ground state HOMO-LUMO (HL) energy shifts $\Delta E_{HL} = E_{HL}^{Pristine} - E_{HL}^{Defect}$ as a function of pristine HL energy, calculated from density functional theory (DFT). The color of the symbols represents the chemical composition of the auxiliary group: Aryl-NH_2/H (red) in Argon and Aryl-NH_2/OH (black) in Air. The symbol shape and fill type represent the defect configuration: *ortho*(+) (Filled Squares), *ortho*(++) (Filled Circles), *ortho*(-) (Filled Triangles), *para*(+) (Open Squares), *para*(++) (Open Circles), and *para*(-) (Open Triangles). The fitted curves represent the same trends as seen in Fig 3b. We show that the deviation between the red and black arises directly from the auxiliary attachment of either H or OH groups. (b) Comparison of the molecular orbital diagram of the pristine and functionalized nanotube by aryl and hydrogen (Aryl-H) and aryl and OH (Aryl-OH) covalently attached to a single carbon-ring at the tube surface in the *ortho*(+) position providing the largest redshift of the lowest exciton. (c) The difference between the HOMO-LUMO energy gaps of the pristine and the functionalized nanotubes (ΔE_{HL}). All calculations are performed using CAM-B3LYP functional and 3-21G basis set using Gaussian16 software package. The results for Aryl-OH are compared for different basis set: 3-21G and the larger mixed 3-21G/6-31G*, where 3-21G is assigned to the SWCNT atoms and 6-31G* to atoms of the functional groups. The results obtained with the mixed basis are expected to be more accurate, although, the extended basis set does not change the qualitative trend of slightly increased HOMO-LUMO gap (by 20-40 meV) of aryl-OH compared to aryl-H.

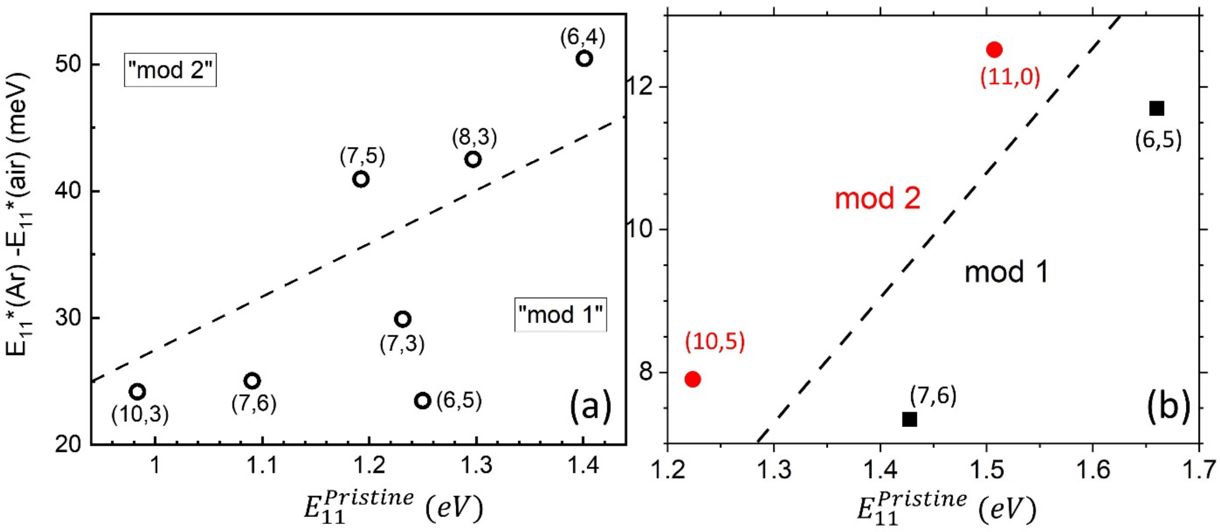

Supplementary Figure 9. (a) The energy differences of E_{11}* emission treated in argon and air for different SWCNT chiralities *vs*. SWCNT E_{11} energy. The "mod 2" nanotube chiralities give slightly larger values of E_{11}* (Ar) – E_{11}* (air) than the "mod 1" nanotubes. (b) Results from ground state density functional theory (DFT) show the same trends in HOMO-LUMO gap energies with respect to the "mod" value of the SWCNT chirality as seen in the E_{11}* emission in (a). These results were obtained using a minimal basis set and without excitonic effects, leading to reduced energetic splitting, which has been previously studied[1].

Supplementary Table 2. Spectral characteristics for E_{11}, E_{11}*, E_{11}** emission of eight different nanotube chiralities

(n,m)	E_{11} (nm)	E_{11}* (nm; air)	E_{11}* (nm; Ar)	E_{11}** (nm; Ar)	Δ E_{11}* (meV; air)	ΔE_{11}* (meV; Ar)	ΔE_{11}** (meV; Ar)
(6,4)	885	1062	1018	1178	234	183	348
(8,3)[2]	956	1128	1086	1250	198	155	305
(6,5)	992	1138	1114	1252	160	137	260
(7,3)	1007	1168	1136	1220	170	140	215
(7,5)	1040	1190	1145	1300	150	109	238
(11,0)	1060		1184	1324		123	233
(7,6)	1137	1275	1243	1366	118	93	183
(10,3)	1261	1415	1377	1440	107	83	122

Supplementary Table 3. Geometrical information for each finite sized pristine SWCNT used in density functional theory calculations[3].

(n,m)	#Atoms	Length (nm)	# Unit Cells	# CH_2 Groups
(6,5)	1114	12.5	3	0
(7,6)	1550	14.8	3	0
(11,0)	1094	10.6	20	4
(10,5)	1438	11.8	10	2

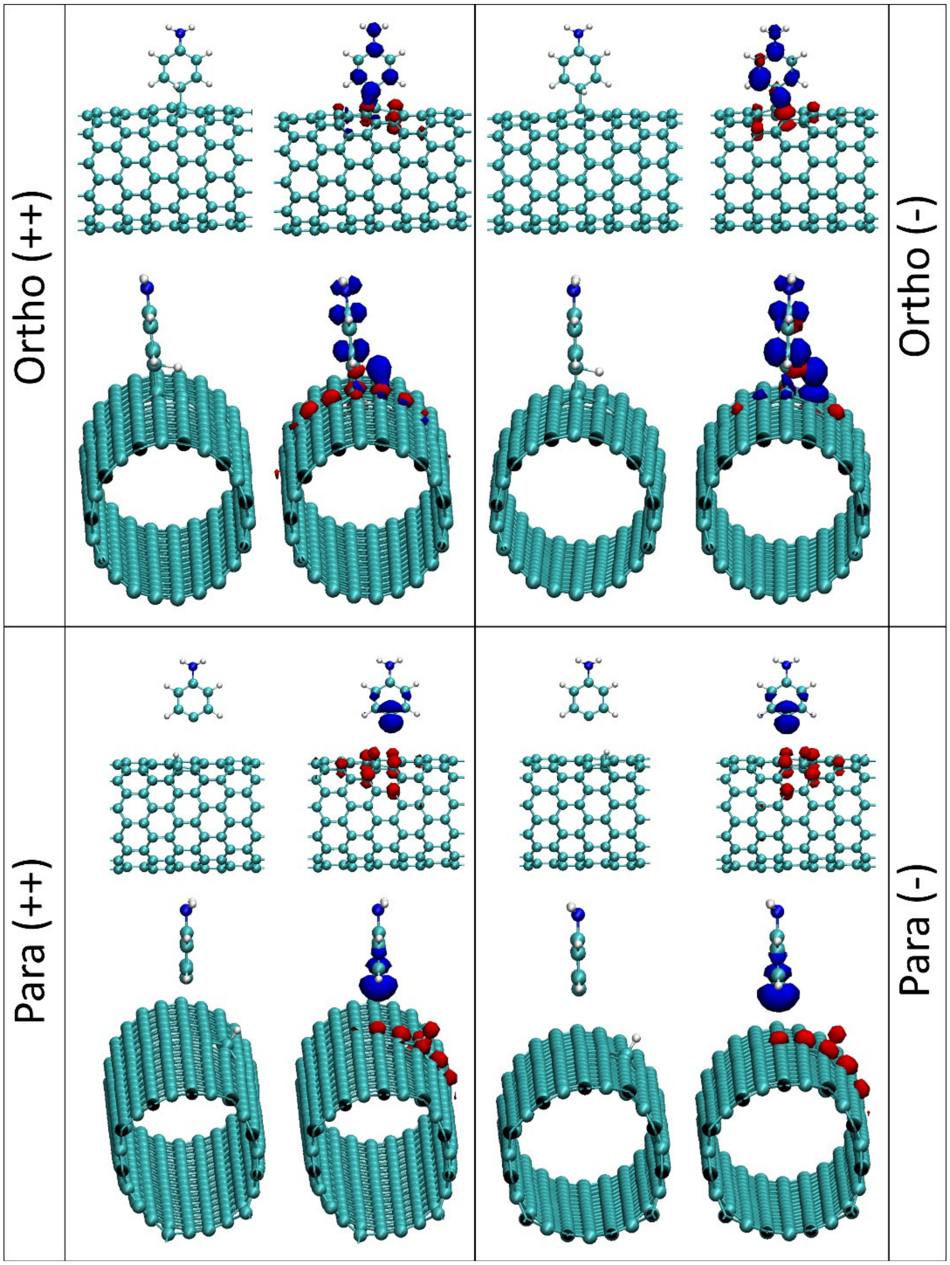

Supplementary Figure 10. Natural spin orbitals (NSOs) were generated from at the transition state geometry for each position shown in Fig. 5c using an extended, finite SWCNT with correct capping scheme (see Supplementary Table 3). The first column of each configuration shows the bare geometry, while the second shows the corresponding NSOs overlayed. The red and blue iso-surfaces correspond to the two NSOs with population equal to 1 and correspond to the diagonalization of the spin density matrix after a ground state calculation with triplet spin multiplicity. In P++, and P-, one can see the spatial separation of the two electrons (i.e., two doublets). In the other, O++ and O-, defect configurations, the two electrons are spatially mixed

and represent more of a triplet state configuration. The natural spin orbitals (NSOs) were calculated using ground state density functional theory information as input (from Gaussian 16 [1]) via the Multiwfn package [2]. The transition state geometries from the nudged elastic band calculations (see methods) were taken and converted to finite sized SWCNTs by replicating the unit cell two times, removing the defect atoms in the outer two cells, and capping with four methylene groups (see Supplementary Table 3). The SWCNT was then optimized using spin-restricted B3LYP/STO-3G, keeping the center 0.5 nm on either side of the defect fixed. This allowed the boundaries between unit cells to relax. Then a single point calculation using spin-polarized B3LYP/STO-3G was completed in the triplet multiplicity.

Supplementary Note 2

Analyses of calculated structural dynamics

The processes of formation of a covalent sp^3-hybridized defect can be described in terms of four key atoms and three interatomic distances between them illustrated in Supplementary Fig. 11. Specifically, a cleavage of C-H bond of aniline is referred in terms of elongation of C_p-H_t distance, while a migration of hydrogen (H_t) from aniline is described in terms of shortening of the C_c-H_t distance with a final formation of the C_c-H_t bond with the nanotube following by a rapid shortening of the C_p-C_c distance resulting in the bond formation between the Aryl-NH_2 and the nanotube.

Supplementary Figures 12 and 13 show the length of C_p-C_c (blue triangles), C_p-H_t (red squares), and C_c-H_t (green circles) interatomic distances along the reaction path resulting in the formation of *ortho* (Supplementary Fig. 12) and *para* defects (Supplementary Fig. 13) calculated for (11,0) SWCNT. The reaction trajectories passing though the transition state (TS) (intermediate #6 in Supplementary Fig. 12) and resulting in ortho-configurations of Aryl-NH_2/H attachments to the (11,0) SWNT are quite similar for both o- and o++ defects. At the earlier stage of the reaction (snapshots from the reactant to the intermediate #5 in Supplementary Fig. 12), a distance from Aryl-NH_2 to H_t (red squares) stays nearly a constant of ~1 Å, while the distance between C_p carbon of aryl and C_c carbon of the SWCNT (blue triangles) systematically decreases from 6.5 Å to 3.5 Å. At the TS (intermediate #6) C_p-C_c bond length (blue triangles) is close to its equilibrium value, while the traveling hydrogen H_t is on the halfway between C_p carbon of aryl (~1.7 Å, red squares) and C_h carbon of the nanotube (~2 Å, green circles). At the last steps of the reaction after TS (the intermediate #7 and the product in Supplementary Fig. 12), the traveling hydrogen completes its departure from aryl and settles at the SWCNT at the *ortho* bonding position.

Analogously to the *ortho* defects, the reaction trajectory for the *para* defects p- is nearly the same as for p++ (Supplementary Fig. 13). However, there is a significant difference in trajectories between *para* and *ortho* defects. For the pathway leading to the para-binding, it takes more steps (more intermediates) for H_t to break of aniline and move towards the C_h of the SWCNT, compared to *ortho* defects. This longer pathway is rationalized by the requirement of a larger distance between C_p and C_h placed at the opposite sides of the carbon ring of the nanotube to provide a *para* attachment of Aryl-NH_2/H to the SWCNT. Therefore, H_t has to move far away from aryl, reaching up to 4 Å of the C_p-H_t distance (intermediate #5 in Supplementary Fig. 13), to end up at the *para* position, than it does for the *ortho* position (~2 Å of the C_p-H_t distance for intermediate #6 in Supplementary Fig. 12). Note that due to a larger mass and a moment of inertia, aryl is much less flexible in its movements compared to H_t, and, therefore, is attaching directly to the closest C_c of the nanotube immediately after H_t is closely approaching the C_h carbon of the nanotube.

Thus, there is a qualitative difference between the nature of TSs and mechanisms of reactions between *para* and *ortho* defects. For *para* defects, the hydrogen H_t is approaching the nanotube first followed by aryl attaching to the nanotube at its TS. In contrast, aryl bounds to the nanotube first followed by H_t attaching to the nanotube at the TS of *ortho* defects. This difference in reaction trajectories requires a significantly higher kinetic energy of H_t to provide *para* defects, compared to the *ortho* defects. This explains rare formation of *para* defects compared to highly probable *ortho* defects at SWCNTs via chemical reactions, as was reported previously[6]. In contrast, photoreactions might provide enough energy to facilitate formations of *para* defects.

To further explore the transition states in *para*-configurations, we carry out two additional calculations. First, we perform spin-restricted DFT based *ab initio* molecular dynamics (AIMD) calculations using intermediate structures obtained from CI-NEB in Supplementary Fig. 13. Such calculations are performed at 100 K with a time step of 1 fs. One finds the smooth connection between initial reactant (CNT + aniline) and the final product (*para*(-) or *para*(++) configuration) along the AIMD trajectories. We then carry out single point energy calculations by spin-polarized DFT for all snapshots along the AIMD trajectories, from which energy profiles for snapshots through singlet and triplet pathways are obtained (Supplementary Fig. 14a). In Fig. 14b, we track three key interatomic distances along the trajectories. Note the energy profiles and evolution of interatomic distances are similar for *para*(-) and *para*(++) configurations, due to the symmetry of

these conformations. We find an increase in total energy of snapshots when the hydrogen starts to get ejected from the aniline molecule and migrates to the tube. Figure 14c shows snapshots along the trajectory for *para*(-) corresponding to the region between images #4 and #6 in Supplementary Fig. 13. Starting from 74 fs (the third snapshot from the left in the second row of Fig. 14c), one finds the total energy of a triplet state is becoming lower than that of a singlet state. This trend remains until 132 fs when the hydrogen migration completes, and the aryl group gets closer to the tube. The TS state predicted by CI-NEB (image #5 in Supplementary Fig. 13 corresponds to the third snapshot from the left in the third row of Fig. 14c.

Second, we perform CI-NEB calculations with 15 images of intermediates (Supplementary Figs. 15 and 16). Since the reaction pathways for *para*(-) and *para*(++) are similar, we only focus on the former to save numerical resources. Our numerical results confirm that the reactant (images # 1-8, 14-15) and the product are in a singlet state, whereas images #9-13 are in a triplet state. The TS state is at image #10 with an energy of 4.944 eV, which is close to the TS energy of 4.943 eV obtained from the CI-NEB calculation with 7 images. The TS structures obtained by 15 images and 7 images are similar, except that the ejected hydrogen is becoming about 0.7 Å closer to the binding site on the tube surface for the former.

We rationalize the obtained results by the difference in the electron-vibrational couplings between triplet and single electronic states. The triplet electronic state has a strong coupling with the vibrational mode corresponding to the stretching of the H-C bond of aryl, compared to the singlet state. This results in a stronger distortion of the C-H bond of aryl in its triplet state leading to the transitional state involving detached H from the aryl group and bonding to the nanotube. Then the aryl radical is attaching to the nanotube in the para position without any significant changes in its location with respect to the CNT with bonded H, due to its large moment of inertia.

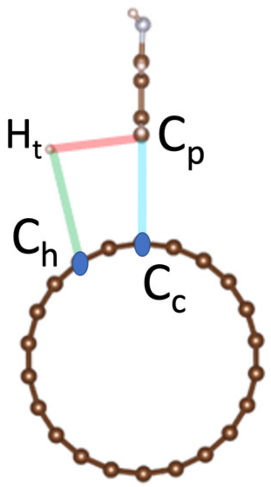

Supplementary Figure 11: Relative position of Aryl-NH_2/H group with respect to (11,0) SWCNT surface is characterized by positions of four key atoms: A "traveling" hydrogen (H_t), a "parent" carbon (C_p) at the aryl-NH_2 initially bonded to H_t, and two carbons of the nanotube converting from sp^2- to sp^3-hybridized bonding due to an adsorption of aryl-NH_2 to the carbon marked as C_c and an adsorption of H_t to the carbon marked as C_h. These key atoms define the following interatomic distances: C_p-C_c showing the distance between two closest carbons of the Aryl-NH_2 and the nanotube (the blue line here and blue triangles in Supplementary Figures 12 and 13), C_p-H_t corresponding to the distance between terminating/traveling hydrogen and the carbon of the aryl-NH_2 (the red line here and red squares in Supplementary Figs. 12 and 13), and C_c-H_t defining the distance between the traveling hydrogen and the carbon of the nanotube to which it will be attached at its final state (the green line here and green circles in Supplementary Figures 12 and 13).

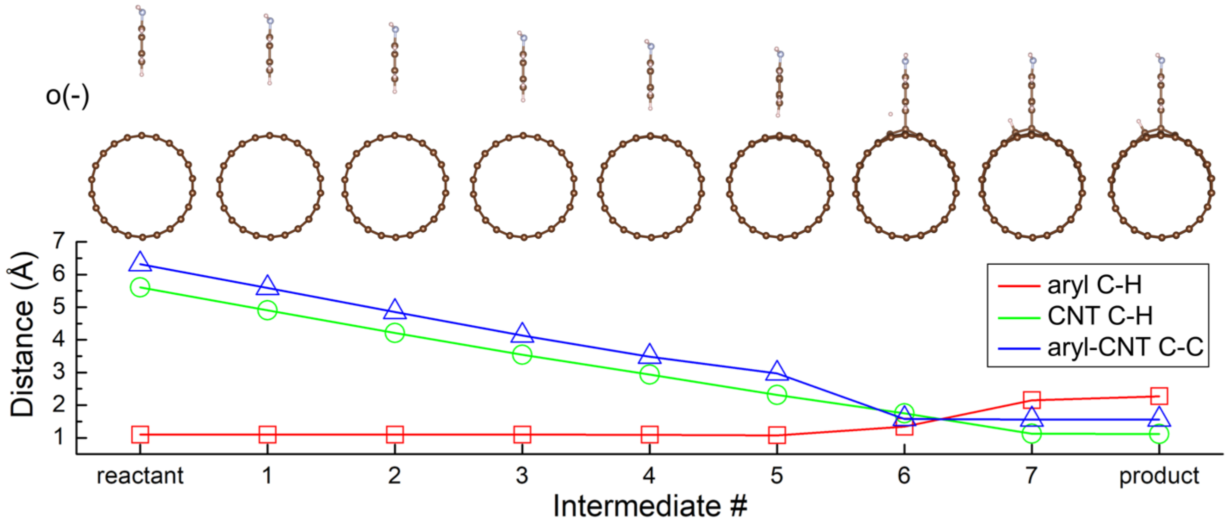

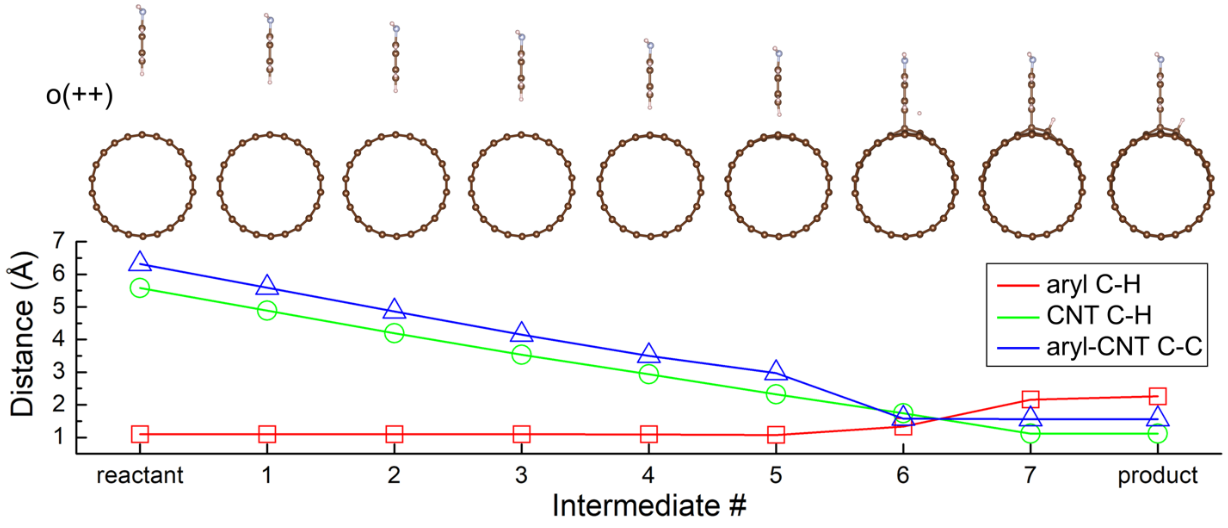

Supplementary Figure 12: A sequence of intermediates between the reactant ((11,0) SWCNT and aniline) and the product (SWCNT with attached Aryl-NH_2/H) for O(-) and O(++) defect configurations. The images of intermediates are shown along the tube axis. Three key interatomic distances along the reaction path correspond to the distance between two closest carbons of the Aryl-NH_2 and the nanotube (blue triangles), the distance between terminating/traveling hydrogen and the carbon of the aryl-NH_2 (red squares), and the distance between traveling hydrogen and the carbon of the nanotube to which it will be attached at its final state (green circles).

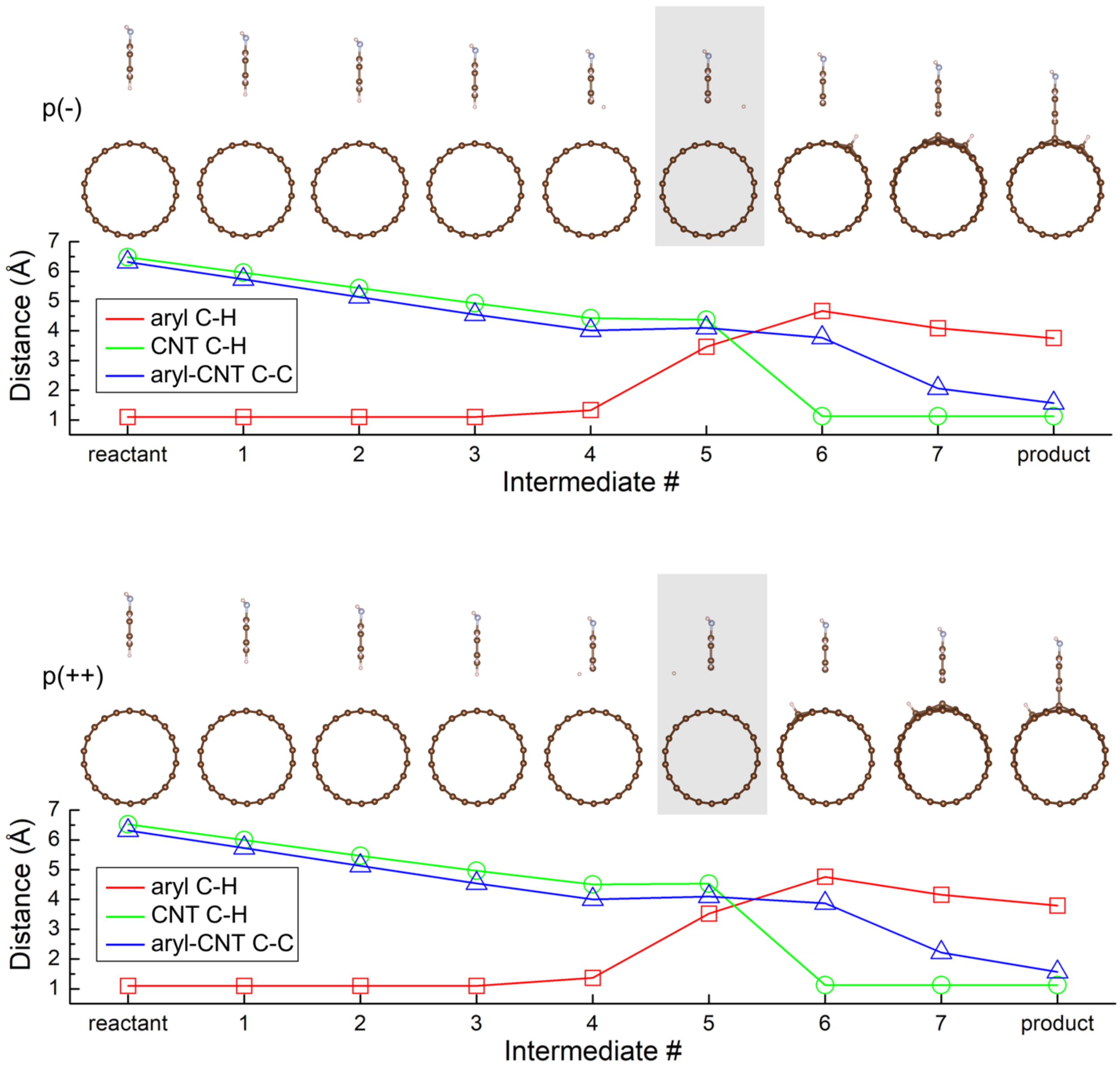

Supplementary Figure 13: A sequence of intermediates between the reactant ((11,0) SWCNT and aniline) and the product (SWCNT with attached Aryl-NH_2/H) for P(-) and P(++) defect configurations. The images of intermediates are shown along the tube axis. Three key interatomic distances along the reaction path correspond to the distance between two closest carbons of the Aryl-NH_2 and the nanotube (blue triangles), the distance between terminating/traveling hydrogen and the carbon of the aryl-NH_2 (red squares), and the distance between traveling hydrogen and the carbon of the nanotube to which it will be attached at its final state (green circles). The snapshot highlighted by grey corresponds to the transition state (intermediate #5). At the intermediate #5,

the bond between the nanotube and aryl is not yet formed and C_p-C_c bond length is far from its equilibrium value (~ 4 Å), while the hydrogen makes a non-trivial path of its migration: Instead of moving along a straight-line towards the SWCNT, it departs far away from both aryl (~4 Å) and the nanotube surface (~5 Å).

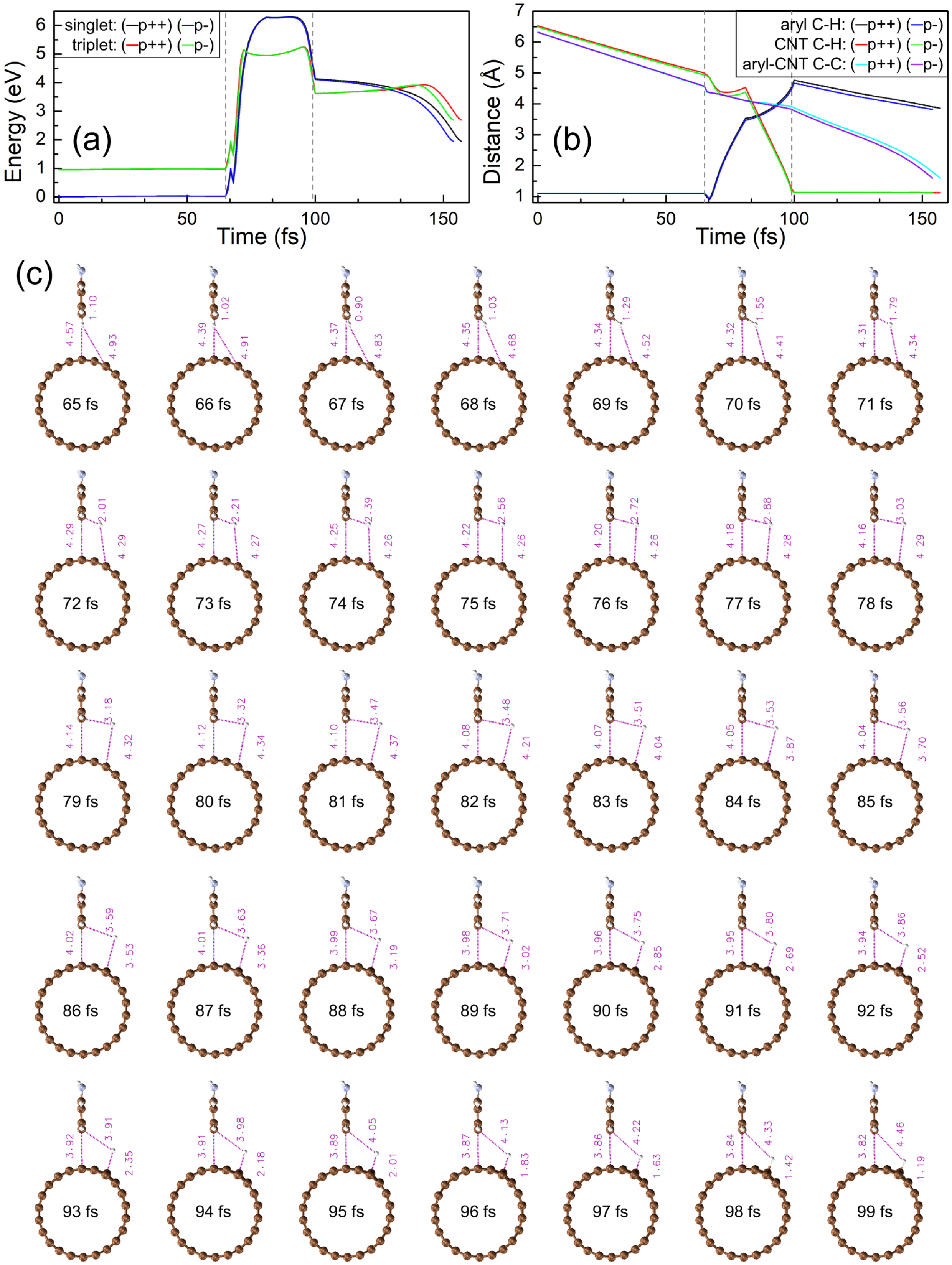
(a)
Energy (eV)
Time (fs)
singlet: (—p++) (—p-)
triplet: (—p++) (—p-)
(b)
Distance (Å)
Time (fs)
aryl C-H: (—p++) (—p-)
CNT C-H: (—p++) (—p-)
aryl-CNT C-C: (—p++) (—p-)
(c)
65 fs
66 fs
67 fs
68 fs
69 fs
70 fs
71 fs
72 fs
73 fs
74 fs
75 fs
76 fs
77 fs
78 fs
79 fs
80 fs
81 fs
82 fs
83 fs
84 fs
85 fs
86 fs
87 fs
88 fs
89 fs
90 fs
91 fs
92 fs
93 fs
94 fs
95 fs
96 fs
97 fs
98 fs
99 fs

Supplementary Figure 14: (a) Calculated energy profiles along the DFT-based AIMD trajectories, which convert the reactant ((11,0) SWCNT and aniline) into the product (SWCNT with attached Aryl-NH_2/H) for *para*(-) and *para*(++) defect configurations. (b) Three key interatomic distances along the trajectories correspond to the distance between two closest carbons of the Aryl-NH_2 and the nanotube, the distance between terminating/traveling hydrogen and the carbon of the aryl-NH_2, and the distance between traveling hydrogen and the carbon of the nanotube to which it will be attached at its final state. (c) The snapshots in the region defined by dashed grey lines in panels (a) and (b) for the AIMD trajectory leading to *para*(-) configuration shown along the tube axis. The AIMD calculations are based on images of intermediates from CI-NEB calculations. The AIMD calculations are performed at 100 K with a time step of 1 fs.

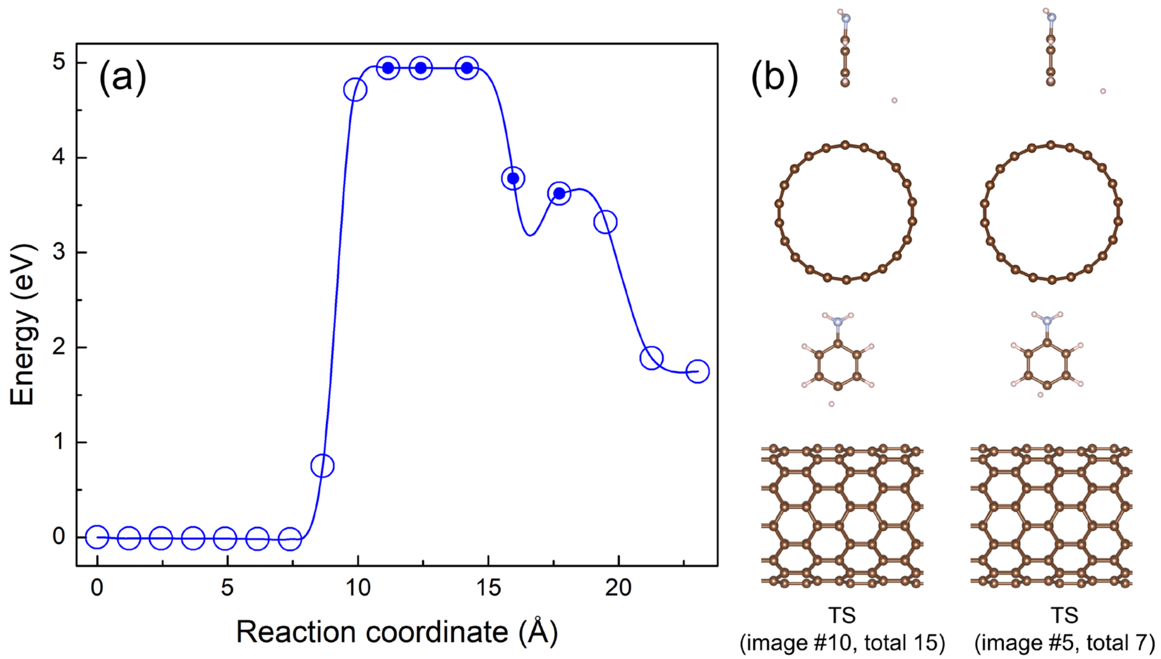

Supplementary Figure 15: (a) Calculated minimum energy path leading to *para*(-) binding configuration by CI-NEB with 15 images of intermediates. Structures with open circles (the reactant, images #1-8, 14-15, and the product) are in a singlet state, whereas those with closed circles (images #9-13) are in a triplet state. (b) comparison of TS states from CI-NEB with 15 images of intermediates and with 7 images of intermediates (Supplementary Figure 13).

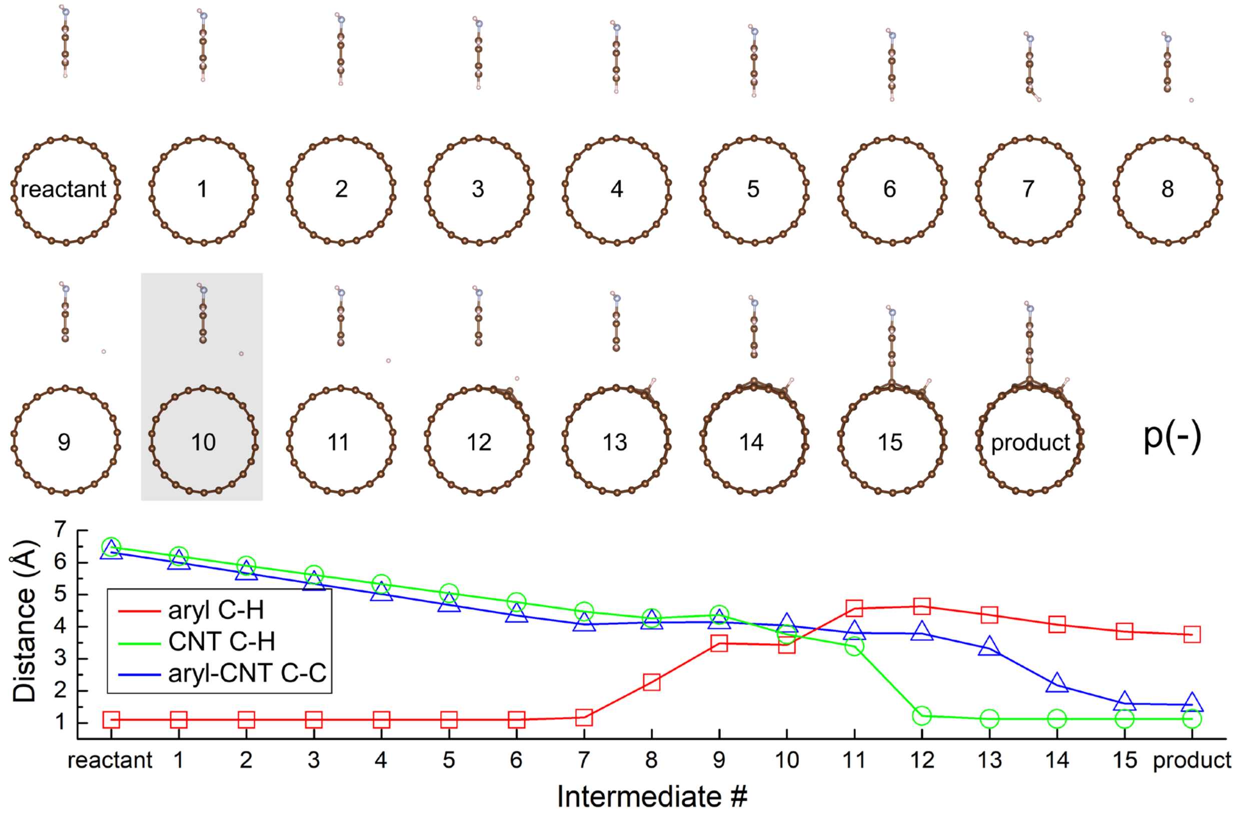

Supplementary Figure 16: A sequence of intermediates between the reactant ((11,0) SWCNT and aniline) and the product (SWCNT with attached Aryl-NH_2/H) for *para*(-) defect configuration. The images of intermediates are shown along the tube axis. Three key interatomic distances along the reaction path correspond to the distance between two closest carbons of the Aryl-NH_2 and the nanotube (blue triangles), the distance between terminating/traveling hydrogen and the carbon of the aryl-NH_2 (red squares), and the distance between traveling hydrogen and the carbon of the nanotube to which it will be attached at its final state (green circles). The snapshot highlighted by grey corresponds to the transition state (intermediate #10).

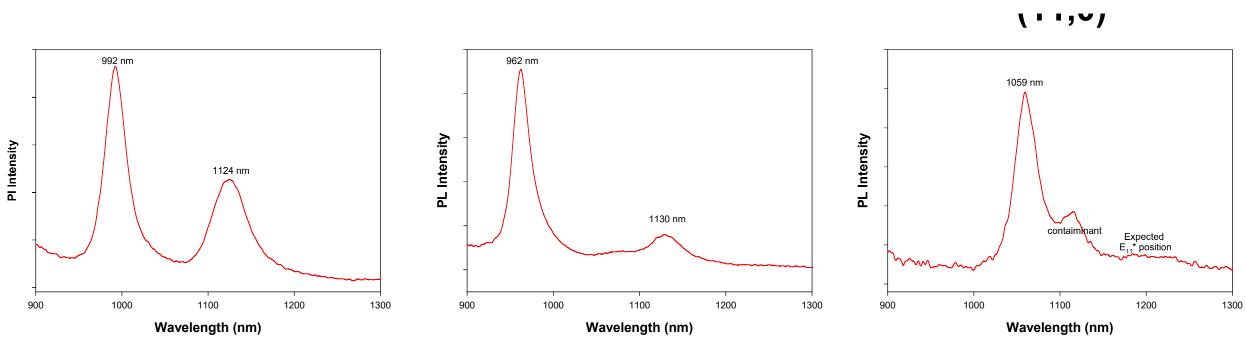

Supplementary Figure 17: A strong chiral angle dependent reaction outcome with iodoaniline in air: a strong E_{11}* emission at 1124 nm (ortho configuration) in near-armchair (6,5) SWCNT becomes weaker in the intermediate case in (8,3) tube at 1130 nm and finally is non-measurable in a zigzag (11,0) tube.

Supplementary References

1 Weight, B. M., Gifford, B. J., Tretiak, S. & Kilina, S. Interplay between Electrostatic Properties of Molecular Adducts and Their Positions at Carbon Nanotubes. *The Journal of Physical Chemistry C* **125**, 4785-4793, (2021).

2 Zheng, Y., Bachilo, S. M. & Weisman, R. B. Photoexcited Aromatic Reactants Give Multicolor Carbon Nanotube Fluorescence from Quantum Defects. *ACS Nano* **14**, 715-723, (2020).

3 Sharma, A., Gifford, B. J. & Kilina, S. Tip Functionalization of Finite Single-Walled Carbon Nanotubes and Its Impact on the Ground and Excited State Electronic Structure. *The Journal of Physical Chemistry C* **121**, 8601-8612, (2017).

4 Frisch, M. *et al.* Gaussian 16 Rev. B. 01. 2016. *Gaussian, Inc. Wallingford CT* (2016).

5 Lu, T. & Chen, F. Multiwfn: A multifunctional wavefunction analyzer. *Journal of Computational Chemistry* **33**, 580-592, (2012).

6 Saha, A. *et al.* Narrow-band single-photon emission through selective aryl functionalization of zigzag carbon nanotubes. *Nature Chemistry* **10**, 1089-1095, (2018).
