# Supplementary material for: Photochemical spin-state control of binding configuration for tailoring organic color center emission in carbon nanotubes
Source: Nat Commun. 2022 Aug 1;13:4439. doi: 10.1038/s41467-022-31921-0 (PMC9343348; doi:10.1038/s41467-022-31921-0)
Supplement: Supplementary file 3 — Description of Additional Supplementary Files [file 41467_2022_31921_MOESM3_ESM.docx]

Supplementary movie 1

Caption: The movie shows formation of ortho binding.

Supplementary movie 2

Caption: The movie shows formation of para binding.

Supplementary Dataset 1

Caption: The dataset contains the ground-state optimized geometries (65 in total) as well as the extended/capped transition state geometries (4 in total) for all chiralities, configurations, and choice of H/OH reported at B3LYP level.

Supplementary Dataset 2

Caption: The dataset contains

1. five long tubes (pristine, o-, o++, p-, p++) for emission in figure 4a

2. five short tubes (one reactant: pristine, and four products: o-, o++, p-, p++) for CI-NEB

3. three short tubes (one initial configuration: pristine, and two final configurations: p- and p++) for AIMD in supplemental Figure S14.
